# Are antidepressants held to a different standard?

**DOI:** 10.1192/bjo.2026.12043

**Published:** 2026-07-17

**Authors:** Ovais Wadoo, Yasser Saeed Khan, Javed Latoo, Abdul Majid Gania, Majid Alabdulla

**Affiliations:** Department of Psychiatry, Hamad Medical Corporation, Doha, Qatar; Department of Psychiatry, Qatar University College of Medicine, Doha, Qatar; Department of Psychiatry, SKIMS Medical College and Hospital, Srinagar, India

**Keywords:** Antidepressive agents, depressive disorder, evidence-based medicine, treatment outcome, clinical decision-making

## Abstract

This Editorial examines the continuing debate over the efficacy and outcomes of antidepressants, focusing on how evidence is interpreted and applied in clinical practice. Although concerns about uncertainty, heterogeneity and contested outcome measures are valid, these challenges are not unique to psychiatry, but are common across medical specialties. The Editorial discusses issues in evidence synthesis, the distinction between statistical significance and clinical relevance, the translation of global trial data into real-world settings and the influence of public and digital discourse. It emphasises balanced interpretation, transparent risk–benefit communication and shared decision-making, to avoid simplistic conclusions about treatment effectiveness.

**Figure:**
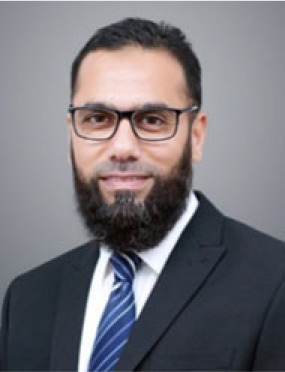

Antidepressants remain among the most widely prescribed psychotropic medications worldwide, yet few treatments in psychiatry have generated such sustained controversy. Debates around uncertainty, heterogeneity of response and interpretation of outcomes remain central and merit careful consideration. Views on antidepressants are further shaped by personal experience, discussions in online forums and influential voices across social and traditional media, contributing to a discourse that is frequently more polarised than the underlying evidence would suggest.^1^ However, these arguments are often presented in isolation, implicitly holding antidepressants to standards rarely applied to other areas of medicine. When placed within a broader medical context, comparable patterns of uncertainty, variable efficacy and reliance on probabilistic outcomes are evident across many widely accepted interventions. Uncertainty is not a weakness of psychiatric science, but an intrinsic characteristic of all areas of medicine that address complex and multifactorial conditions.^2^ Treatment effects are probabilistic rather than absolute, both in psychiatry and across the broader medical field. Outcomes are influenced by multiple interacting factors, including biological variability, population heterogeneity, differences in underlying disease mechanisms, comorbidity, contextual influences, adherence, limitations of outcome measures and restricted follow-up durations. These features are common across medical specialties.^2^ Navigating uncertainty is therefore a central component of evidence-based practice.^3^ Clinicians routinely make decisions under conditions of incomplete or evolving information, integrating available evidence with clinical judgement.^4^ Evidence-based frameworks do not eliminate uncertainty, but provide a structured approach for its management.^3^

The debate surrounding antidepressant efficacy should therefore be understood as part of a broader challenge across medicine rather than evidence that psychiatry occupies an anomalous position. This article examines evidence synthesis, clinical interpretation, global context and the influence of public discourse, alongside emphasising balanced, context-sensitive appraisal.

## Evidence synthesis and heterogeneity

Meta-analyses and systematic reviews aim to reduce uncertainty by pooling data, yet they introduce interpretative challenges. Studies differ in design, populations, comparators and outcomes. Such heterogeneity reflects the limits of interpreting complex clinical evidence rather than deficiencies in scientific reasoning.^5,6^ Similar debates occur across medical specialties when treatments act on complex, multifactorial conditions, where benefits are probabilistic, accrue over time and may involve intermediate or risk-based outcomes rather than cure.^7^ Empirical analyses indicate that antidepressant effect sizes fall within ranges observed in several general medical interventions. These include medications prescribed to reduce cardiovascular risk, manage metabolic disease or prevent long-term complications, where benefits are often incremental and accrue at a population level rather than guaranteeing improvement for every individual patient.^7^ However, such comparisons require careful interpretation. Unlike many chronic medical conditions indexed to biological markers or long-term risk modification, antidepressant treatment is primarily directed toward symptomatic remission and functional recovery. The challenge, therefore, is not that antidepressants are uniquely ineffective, but that their benefits must be interpreted within a framework of heterogeneous and probabilistic response, where remission is the goal, but outcomes vary across individuals. A clinically salient expression of this variability is treatment-resistant depression, typically defined by inadequate response to successive antidepressant trials. Although frequently invoked in critiques of antidepressant efficacy, treatment-resistant depression more plausibly represents the upper end of a response distribution shaped by illness severity, chronicity and biological variability.^8^ As in other areas of medicine, non-response in a subset of patients does not negate average treatment benefit, but reflects the distributional nature of therapeutic effects in heterogeneous populations. By placing antidepressants within this wider medical context, psychiatry is not unfairly singled out as less scientific or less evidence-based. Instead, it is understood as operating under the same epistemic and methodological constraints that affect evidence generation and interpretation across medicine.

## Statistical significance and clinical relevance

A frequent source of confusion arises when statistical significance is mixed up with clinical relevance. Although small effect sizes are often considered clinically insignificant, even modest benefits may be meaningful, particularly in chronic or recurrent mental disorders where small improvements can have a significant impact on quality of life. The interpretation of efficacy of antidepressants is often influenced by variation in diagnostic systems and the symptom-based nature of psychiatric assessment. Differences between DSM and ICD criteria, as well as reliance on rating scales rather than biological markers, can affect case identification, outcome measures and treatment response. These factors may also contribute to discrepancies between clinician- and patient-reported outcomes in trials. In addition, the stigma surrounding psychiatric disorders can amplify scrutiny of effect sizes and reduce tolerance for probabilistic benefit.^9^ Together, these considerations reflect methodological and contextual influences on interpretation, rather than deficiencies in the underlying evidence.

Network meta-analyses demonstrate that commonly used antidepressants are more effective than placebo in major depressive disorders.^10^ Differences between individual agents are generally small, a pattern also observed across pharmacotherapy. Clinical decision-making therefore extends beyond symptom scales, to include number needed to treat, functional outcomes, tolerability and patient-reported benefit. The real-world effectiveness may be further influenced by dose–response relationships and optimisation of treatment.^11^ Placebo response and expectancy effects discussions are also not unique to psychiatry and apply to many areas of medicine where subjective outcomes play a role.^12^ Treatment outcomes and efficacy of antidepressants should therefore be assessed with the same standards applied to pharmacological treatments in other medical specialties. Applying more stringent or inconsistent criteria risks reinforcing the perception that psychiatric treatments are supported by a weaker or less consistent evidence base.

## Global evidence, context and implementation

Systematic reviews are predominantly derived from high-income settings, limiting generalisability.^6,13^ Variations in infrastructure, access and population characteristics introduce contextual modifiers often not captured in pooled estimates. Across low- and middle-income settings, outcomes are strongly shaped by social determinants, including socioeconomic adversity, stigma and workforce limitations.^14,15^ These factors affect adherence, continuity of care and monitoring, thereby moderating translation of efficacy into effectiveness. This distinction between efficacy and effectiveness is consistent across medicine. Antidepressant benefit in practice depends on implementation factors such as follow-up, dose optimisation and integration with psychosocial care. Evidence suggests collaborative and task-shared care models can enhance outcomes.^14,15^

From a policy perspective, interpretation of evidence must incorporate feasibility, cost-effectiveness and equity alongside efficacy. Outcome frameworks extending beyond symptom reduction, including functioning and quality of life, may better capture meaningful benefits. Antidepressants are therefore best understood as one component within broader systems of care.

## Public discourse, digital media and trust in evidence

Perceptions of antidepressants vary across stakeholders. Clinicians prioritise guidelines, patients emphasise lived experience and public views are increasingly shaped by media narratives.^16,17^

The enduring stigma associated with mental disorders also shapes perceptions. Although illness is predominantly conceptualised biologically across many areas of medicine, mental disorders are understood through an overlap of moral, psychological and social frameworks. As a result, pharmacological treatments in psychiatry may attract greater scepticism and face reduced tolerance for modest benefits. Uncertainty in psychiatric evidence may therefore be interpreted more critically than in other specialties.

Digital platforms can amplify simplified narratives, overshadowing methodological nuance.^2,18^ Psychiatrists and other clinicians therefore have a shared responsibility to engage constructively in public and digital spaces, communicate evidence clearly and acknowledge uncertainty without undermining trust. The lack of discharging this important professional responsibility risks handing over public understanding to misinformation, wrong perceptions and oversimplification.

Shared decision-making provides a practical framework to navigate uncertainty, requiring clinicians to integrate evidence with patient values, preferences and circumstances, and to support decisions that are both scientifically grounded and personally meaningful.

## The way forward

A pragmatic approach requires moving beyond binary interpretations of efficacy toward context-sensitive application of evidence. Clinical decisions should integrate aggregate data with individual presentation, treatment history and patient preferences, recognising that benefit is variable and may emerge over time. Iterative assessment, including modification or discontinuation where response is limited, remains central to prescribing. Rather than eliminating uncertainty, clinical practice proceeds through its structured management, supported by ongoing evaluation and transparent communication. This includes acknowledging evidentiary limits, incorporating patient preference and adapting interventions to local contexts. Within this framework, antidepressants are best viewed not in isolation, but as part of a multimodal strategy in which pharmacological effects interact with psychological, social and systemic factors. Such an approach avoids both overstatement and dismissal of treatment effects and aligns interpretation with broader medical standards, where modest and probabilistic benefits remain clinically meaningful when balanced against illness burden and available alternatives.
